# Ability of the Encephalitic Arbovirus Semliki Forest Virus To Cross the Blood-Brain Barrier Is Determined by the Charge of the E2 Glycoprotein

**DOI:** 10.1128/JVI.03645-14

**Published:** 2015-05-13

**Authors:** Mhairi C. Ferguson, Sirle Saul, Rennos Fragkoudis, Sabine Weisheit, Jonathan Cox, Adjanie Patabendige, Karen Sherwood, Mick Watson, Andres Merits, John K. Fazakerley

**Affiliations:** a The Pirbright Institute, Pirbright, Woking, United Kingdom; b Institute of Technology, University of Tartu, Tartu, Estonia; c The Roslin Institute, University of Edinburgh, Easterbush, Midlothian, United Kingdom; d Institute of Infection and Global Health, University of Liverpool, Liverpool, United Kingdom; e Edinburgh Genomics, The Roslin Institute and Royal (Dick) School of Veterinary Studies, University of Edinburgh, Easterbush, Midlothian, United Kingdom

## Abstract

Semliki Forest virus (SFV) provides a well-characterized model system to study the pathogenesis of virus encephalitis. Several studies have used virus derived from the molecular clone SFV4. SFV4 virus does not have the same phenotype as the closely related L10 or the prototype virus from which its molecular clone was derived. In mice, L10 generates a high-titer plasma viremia, is efficiently neuroinvasive, and produces a fatal panencephalitis, whereas low-dose SFV4 produces a low-titer viremia, rarely enters the brain, and generally is avirulent. To determine the genetic differences responsible, the consensus sequence of L10 was determined and compared to that of SFV4. Of the 12 nucleotide differences, six were nonsynonymous; these were engineered into a new molecular clone, termed SFV6. The derived virus, SFV6, generated a high-titer viremia and was efficiently neuroinvasive and virulent. The phenotypic difference mapped to a single amino acid residue at position 162 in the E2 envelope glycoprotein (lysine in SFV4, glutamic acid in SFV6). Analysis of the L10 virus showed it contained different plaque phenotypes which differed in virulence. A lysine at E2 247 conferred a small-plaque avirulent phenotype and glutamic acid a large-plaque virulent phenotype. Viruses with a positively charged lysine at E2 162 or 247 were more reliant on glycosaminoglycans (GAGs) to enter cells and were selected for by passage in BHK-21 cells. Interestingly, viruses with the greatest reliance on binding to GAGs replicated to higher titers in the brain and more efficiently crossed an *in vitro* blood-brain barrier (BBB).

**IMPORTANCE** Virus encephalitis is a major disease, and alphaviruses, as highlighted by the recent epidemic of chikungunya virus (CHIKV), are medically important pathogens. In addition, alphaviruses provide well-studied experimental systems with extensive literature, many tools, and easy genetic modification. In this study, we elucidate the genetic basis for the difference in phenotype between SFV4 and the virus stocks from which it was derived and correct this by engineering a new molecular clone. We then use this clone in one comprehensive study to demonstrate that positively charged amino acid residues on the surface of the E2 glycoprotein, mediated by binding to GAGs, determine selective advantage and plaque size in BHK-21 cells, level of viremia in mice, ability to cross an artificial BBB, efficiency of replication in the brain, and virulence. Together with studies on Sindbis virus (SINV), this study provides an important advance in understanding alphavirus, and probably other virus, encephalitis.

## INTRODUCTION

Viral encephalitis is a major cause of morbidity and mortality globally. The brain is separated from the blood by the blood-brain barrier (BBB), which prevents the entry of plasma components. Some viruses have the ability to cross the BBB and are neuroinvasive; examples include West Nile virus, Japanese encephalitis virus (JEV), and tick-borne encephalitis virus (TBEV). Other viruses, such as maedi-visna and human immunodeficiency virus (HIV), can enter the central nervous system (CNS) within invading infected cells. Viruses such as Venezuelan equine encephalitis virus (VEEV), poliomyelitis virus, and rabies virus can enter the CNS along nerves (1). For viruses that cross the BBB, little is known about the molecular determinants that confer a neuroinvasive phenotype. Many arboviruses of the Togaviridae, Bunyaviridae, and Flaviviridae families cause a plasma viremia, which can lead to encephalitis. SFV, an Old World alphavirus (Togaviridae), provides a well-characterized model system to study neuroinvasion and acute virus encephalitis.

Following intraperitoneal (i.p.) inoculation into adult mice, SFV establishes a high-titer plasma viremia and enters the brain by passage across the BBB (1). Both well-studied biological isolates SFV L10 and SFV A7(74) are rapidly neuroinvasive. In contrast, virus derived from the molecular clone SFV4 is not (2). L10 and A7(74) both replicate in peripheral tissues, producing a high-level blood viremia. This viremia is controlled by the interferon response and then by antibodies (3). Infectious virus is detectable in the brain between 2 and 10 days postinfection (4). Electron microscopy studies suggest this is by passage across cerebral endothelial cells (5). On entering the brain, L10 and A7(74) initially replicate in perivascular oligodendrocytes and neurons (6–8). From these initial foci, L10 rapidly disseminates throughout the brain, resulting in a fatal panencephalitis (4). In contrast, replication of A7(74) in adult mice is restricted to the original foci of infection, this virus is cleared by the immune response, and infection is avirulent (9). Studies in our laboratory suggest that i.p. infection with low-dose SFV4 produces a lower titer viremia than L10 and that for most infected mice, no virus is detectable in the brain. In contrast, following intracerebral (i.c.) or intranasal (i.n.) inoculation, SFV4 disseminates rapidly throughout the brain, as observed with L10 virus (9). As SFV L10 and SFV4 are closely related variants, these differences in plasma viremia and neuroinvasiveness provide an opportunity to determine the genetic determinants of the phenotypes.

L10 was isolated from a pool of 130 Aedes africanus mosquitoes captured in the Semliki Forest, Uganda (10). L10 was passaged eight times by i.c. inoculation in adult mouse brains, followed by two i.c. passages in neonatal mouse brains (11). Thereafter, the passage history of this virus varied between the laboratories to which aliquots were dispersed. One aliquot was sent from the Bradish laboratory in Porton Down, United Kingdom, to the Webb laboratory in London, United Kingdom. It then was passaged twice in chicken embryo fibroblasts and in 1980 was aliquoted and stored frozen at −80°C. One of these aliquots was used in this study. SFV prototype virus was isolated from the same mosquito sample as L10 and passaged 4 times by i.c. inoculation in adult mice (11). Following dispersion to other laboratories, the passage histories of this virus are not recorded. The SFV prototype was one of the first viruses to be sequenced (12, 13). Molecular clones of the structural and nonstructural regions of the genome were generated from a stock of prototype virus at the Karolinska Institute, Sweden. From these a full-length molecular clone was generated which was able to produce propagating virus. This infectious cDNA (icDNA) clone and the virus derived from it were designated SFV4 (14).

To identify the molecular determinants responsible for the phenotypic differences observed between SFV4 and L10, the consensus nucleotide sequence of the L10 stock was obtained using high-throughput sequencing and compared to the published sequence of SFV4. Twelve nucleotide differences were identified, six of which generated amino acid residue changes. The six nonsynonymous differences were reverse engineered into the SFV4 icDNA. This was used to generate a neuroinvasive virus designated SFV6. Further investigation demonstrated that changes in the virus envelope E2 protein determined the level of plasma viremia and the ability of virus to cross the BBB.

## MATERIALS AND METHODS

### Cells, plasmids, and viruses.

All cell lines were maintained at 37°C with 5% CO_2_. BHK-21 cells (ATCC) were grown in Glasgow's minimal essential medium (GMEM; Life Technologies) supplemented with 10% newborn calf serum (NBCS), 10% tryptose phosphate broth (TPB; Life Technologies), and 100 U/ml penicillin–100 μg/ml streptomycin (Pen/Strep; Life Technologies). NIH 3T3 cells (ATCC) were grown in Dulbecco's modified Eagle medium (DMEM; Life Technologies) supplemented with 10% fetal calf serum (FCS) and Pen/Strep. CHO-K1 (ECACC) and pgsA-745 cells (ATCC) were grown in Kaighn's modification of Ham's F-12K medium (Life Technologies) supplemented with FCS and Pen/Strep. Immortalized human brain endothelial cells (HBECs) were kindly provided by Georges Grau (University of Sydney Medical School). HBECs were grown in collagen-coated T75 flasks and maintained in DMEM (Life Technologies) containing 2 mM etamine, Pen/Strep, 550 nM hydrocortisone (Sigma), 10% FCS, and human recombinant epidermal growth factor (First Link United Kingdom). Primary human astrocytes (catalogue number 1800) were purchased from ScienCell Research Laboratories, USA, and were used at passages 2 to 5. These primary human astrocytes were grown in T75 flasks coated with poly-d-K (Sigma) and were maintained in DMEM-Ham's nutrient mixture F12 (Sigma) containing 10% FCS, 2 mM etamine, and 100 Pen/Strep.

SFV L10 originally was obtained from H. E. Webb, St. Thomas Hospital, London, England, who received it from Claude Bradish, Porton Down, England. In 1980, prior to aliquoting and storing at −80°C, this virus was passaged twice in chicken embryo fibroblasts. Virus from this vial was amplified by a single passage in BHK-21 cells prior to sequencing.

The full-length infectious cDNA (icDNA) clone of SFV4, pSP6-SFV4, was engineered by Peter Liljeström and was used to construct a stabilized infectious SFV plasmid, pCMV-SFV4, as previously described (15). All recombinant clones generated were engineered from the molecular clone pCMV-SFV4. To obtain the infectious clone of SFV6 (pCMV-SFV6), the region containing the 5 nonsynonymous point mutations revealed in the structural region of the SFV genome was obtained as synthetic DNA (Genewiz) and used to replace the corresponding fragment of the pCMV-SFV4 vector utilizing available restriction sites. The change resulting in E1384A substitution in nsP3 was introduced by site-directed PCR mutagenesis. Virus chimeras were generated by exchange of fragments between pCMV-SFV6 and pCMV-SFV4. The sequences of all of the constructs were verified by control restrictions and sequence analysis. Sequences are available from the authors upon request.

All viruses were rescued from respective icDNA clones as previously described (15). Briefly, 5 μg of icDNA was transfected into BHK-21 cells by electroporation (220 V, 975 μF; one pulse in a cuvette with a 4-mm electrode cap). Transfected cells were incubated at 37°C, and virus stocks were collected at 24 to 48 h postinfection (hpi) and subsequently purified by ultracentrifugation through a sucrose cushion (20%). Concentrated virus stocks were resuspended in Tris-acetate-EDTA (TAE) buffer and titrated by plaque assay on BHK-21 cells using an carboxymethyl cellulose–GMEM overlay containing 2% NBCS. The L10 strain of SFV was propagated as previously described for A7(74) (4). Virus was purified and titrated by following the method described for the viruses derived from icDNA.

### High-throughput sequencing.

Approximately 6 × 10^5^ NIH 3T3 cells grown in 6-well plates were infected with SFV L10 at a multiplicity of infection (MOI) of 10. After 8 h, total RNA was extracted using the RNeasy kit (Qiagen) according to the manufacturer's instructions. RNA integrity and concentration were measured by the RNA 6000 Nano assay (Agilent Technologies). The consensus sequence of L10 was generated by sequencing on an Illumina (Solexa) platform using paired-end sequencing. The paired-end reads subsequently were mapped against the reference genome AY112987 using the software novoalign (http://www.novocraft.com/products/novoalign/) (GenBank accession number KP271965). To identify any nucleotide differences between the sequences of the L10 consensus sequence and the published sequence of SFV4 (GenBank accession number KP699763), the sequences were compared using Jalview 2.7 (16).

To investigate the prominent genetic variants within the L10 stock, the L10 reads generated by high-throughput sequencing were mapped to the reference genome using Bowtie2 version 2.1.0 (17) and SAMtools (18) to produce bam files. These files were analyzed in Piledriver (https://github.com/arq5x/piledriver/archive/master.zip) to determine the number of nucleotides possible at each position. A single-nucleotide polymorphism (SNP) was called if the incidence of the prevalent nucleotide occurring at a specific position was between 15% and 85%.

### Mouse infections.

Mouse infections were performed as previously described (2). Three- to 4-week-old BALB/c mice were purchased from Harlan Laboratories, United Kingdom. Mice were housed in the Infectious Diseases Animal Unit, College of Medicine & Veterinary Medicine, University of Edinburgh, United Kingdom, under specific-pathogen-free and environmentally enriched conditions with food and water supplied *ad libitum*. All studies were approved by the University of Edinburgh Ethical Review Committee and were carried out under the authority of a United Kingdom Home Office license. Groups (*n* = 6) of 4- to 5-week-old mice were inoculated i.p. with 0.1 ml PBSA (phosphate-buffered saline [PBS] with 0.75% bovine serum albumin [BSA]) containing 5,000 PFU of virus or i.c. (*n* = 5) with 20 μl PBSA containing 1,000 PFU of virus. Groups of mice were euthanized on postinfection day (PID) 1 or 3; blood samples and brains were collected for analysis. i.p.-inoculated mice (*n* = 8) were monitored twice daily for up to 2 weeks. All mice inoculated i.c. were euthanized at 30 hpi. Half of each brain was stored in fixative for immunohistochemistry. The other half was homogenized in PBSA and used for virus titration. Virus titers in both the blood and the brain samples were determined by plaque assay on BHK-21 cells, as described previously (19). To assess virulence, groups of mice (*n* = 8) were monitored twice daily for up to 2 weeks. Avirulence was defined as at least 7/8 mice surviving for 2 weeks. Virulence was defined as at least 7/8 mice reaching clinically defined endpoints within 1 week.

### *In situ* hybridization.

To observe virus distribution in the brain, after immersion fixation in 10% phosphate-buffered formal saline, half brains were embedded in paraffin and 5-μm sections cut onto poly-l-K-coated (Sigma) or Biobond-coated (British BioCell International) glass slides. ^35^S-labeled riboprobes were transcribed *in vitro* with T7 RNA polymerase from HincII-linearized pGEM1-SFV (4). As a control, some sections were hybridized with a riboprobe to the P1 region of the unrelated Theiler's virus. Probes were hydrolyzed in 0.04 M NaHCO_3_ for 30 min at 60°C prior to use. *In situ* hybridization was carried out as described previously (4). Autoradiographic images of sections were produced by exposure of air-dried sections to Hyperfilm bmax (GE Healthcare). Sections subsequently were dipped in photographic emulsion (LM-1; diluted with 0.66 M ammonium acetate; GE Healthcare) and exposed, usually for 7 days, at 4°C.

### RNA extraction, reverse transcription, PCR, and real-time PCR.

Total RNA was extracted from cell monolayers, and viral RNA was extracted from 100 μl of virus stock using the RNeasy kit (Qiagen) according to the manufacturer's instructions. Total RNA was extracted from 250 μl of cell supernatant using TRIzol-LS reagent (Ambion) according to the manufacturer's instructions. Total RNA was extracted from mouse brains using the RNeasy lipid tissue kit (Qiagen). The RNA then was converted into cDNA using SuperScript III reverse transcriptase (Invitrogen) with oligo(dT)_15_ (Promega) or a first-strand cDNA synthesis kit (Thermo Scientific) with random hexamer primer by following the manufacturer's instructions. Specific regions of the SFV genome, including sequences encoding nsP3, capsid protein, and E2, were amplified by PCR using GoTaq DNA polymerase (Promega) or Phusion polymerase (Thermo Scientific). The PCR products were run on a 1% TAE–agarose gel, and the amplicons were extracted using the High Pure PCR product purification kit (Roche) according to the manufacturer's instructions and analyzed by Sanger sequencing.

Real-time PCR was performed to ensure that virus titers determined by plaque assay correlated with genomic RNA copy number. Reactions were performed using either a ViiA 7 real-time PCR system (Life Technologies) or 7900HT fast real-time PCR system (Life Technologies). For the ViiA 7 real-time PCR system, the master mix contained 0.6 μl of 10 μΜ each primer, 10 μl of 2× FastStart universal SYBR master (Rox) (Roche Applied Science), and 3.8 μl of water. Five microliters of a 1:1,000 dilution of template was added to this. When using the 7900HT fast real-time PCR system, the 10-μl reaction volume contained 0.8 μΜ of each primer, 2 μl of 5× HOT FIREPol EvaGreen quantitative PCR mix (Solis Biodyne), and 2 μl (1/10) of the synthesized cDNA. Each reaction was run in triplicate. All primer sequences are available on request.

### Heparan sulfate interaction studies.

CHO-K1 and glycosaminoglycan (GAG)-deficient pgsA-745 cells seeded in 12-well plates were infected with virus at an MOI of 0.0001 in PBSA for 1 h at room temperature. Cell monolayers then were washed twice with PBS and overlaid with an agarose–GMEM overlay supplemented with 2% FCS. After 3 days at 37°C, cells were fixed and titers calculated as described for other plaque assays.

To remove heparan sulfate from the surface of BHK-21 cells, cells were seeded in 12-well plates and then incubated with 0.5 U/ml of Heparinase I (Sigma) or BSA as a control for 30 min at room temperature with constant agitation. Subsequently, the mixture was replaced with virus in PBSA and incubated for a further 30 min at room temperature with constant agitation. Monolayers then were washed twice with PBS and overlaid with an agarose–GMEM overlay supplemented with 2% FCS. After 2 days at 37°C, cells were fixed and titers determined as described for plaque assays.

For heparin competition assays, virus was incubated with 200 μg/ml of heparin (AppliChem) or BSA as a control for 30 min on ice with constant rotation. BHK-21 cells were exposed to the virus-heparin mix for 30 min on ice, the inoculum was removed, the monolayers were washed twice with PBS, and an agarose–GMEM overlay supplemented with 2% FCS was added. After 2 days at 37°C, cells were fixed and titers determined as described for plaque assays.

### Establishment of the *in vitro* human blood-brain barrier model.

Astrocytes were grown in poly-d-K-coated 12-well plates at 10^5^ cells/well and allowed to grow to confluence. HBEC-5i cells were seeded on collagen-coated Corning Transwell-Clear inserts (12 mm diameter, 0.4 μm pore size) at 10^5^ cells/insert and were transferred to a 12-well plate containing confluent astrocytes. Experiments were performed when HBECs were confluent, typically within 3 days of seeding, and when transendothelial electrical resistance (TEER) across the monolayer was over 60 Ω · cm^2^.

### Transendothelial electrical resistance measurements.

TEER across the BBB model was determined using STX100C electrodes connected to an EVOM2 epithelial voltohmmeter (World Precision Instruments, Hertfordshire, United Kingdom) as described previously (20). The resistance of cells grown on Transwell filter inserts was corrected for resistance across an empty collagen-coated Transwell insert and multiplied by surface area to give TEER in ohms per square centimeter.

### Infecting the BBB model.

The *in vitro* human BBB model was infected with SFV variants (SFV6 and SFV6-162K) from the luminal side at an MOI of 0.1 for 1 h to allow virus to absorb before the addition of fresh media. Virus diluent media (DMEM with 2% FBS) was used for mock-infected controls. TEER was measured at 6 and 8 hpi. Supernatant samples from the abluminal side were taken at 6 and 8 hpi to determine virus titer by plaque assay as described previously (21). All experiments were repeated at least three times (*n* = 3).

### Statistical analysis.

Statistical analysis was carried out using GraphPad Prism. Data were analyzed with the nonparametric Mann-Whitney test (*P* ≤ 0.05) for mouse studies or the Student *t* test (*P* ≤ 0.05).

### Nucleotide sequence accession numbers.

Newly determined sequences were deposited in GenBank under accession numbers KP271965, KP699763, and KT009012.

## RESULTS

The genomes of RNA viruses are known to be highly variable: these viruses generally exist as a mixed population of genotypes. Consistent with this, variable results have been reported between laboratories working with similarly named strains of SFV. These virus stocks have varied in their passage history and may, as a result, be genetically different. Here, we have used a stock of L10 virus of known provenance and compared it to virus derived from the first molecular clone of SFV, SFV4, to determine the basis for their differences in phenotype.

### SFV4 and L10 differ in their level of viremia and ability to enter the CNS in mice.

**FIG 1:**
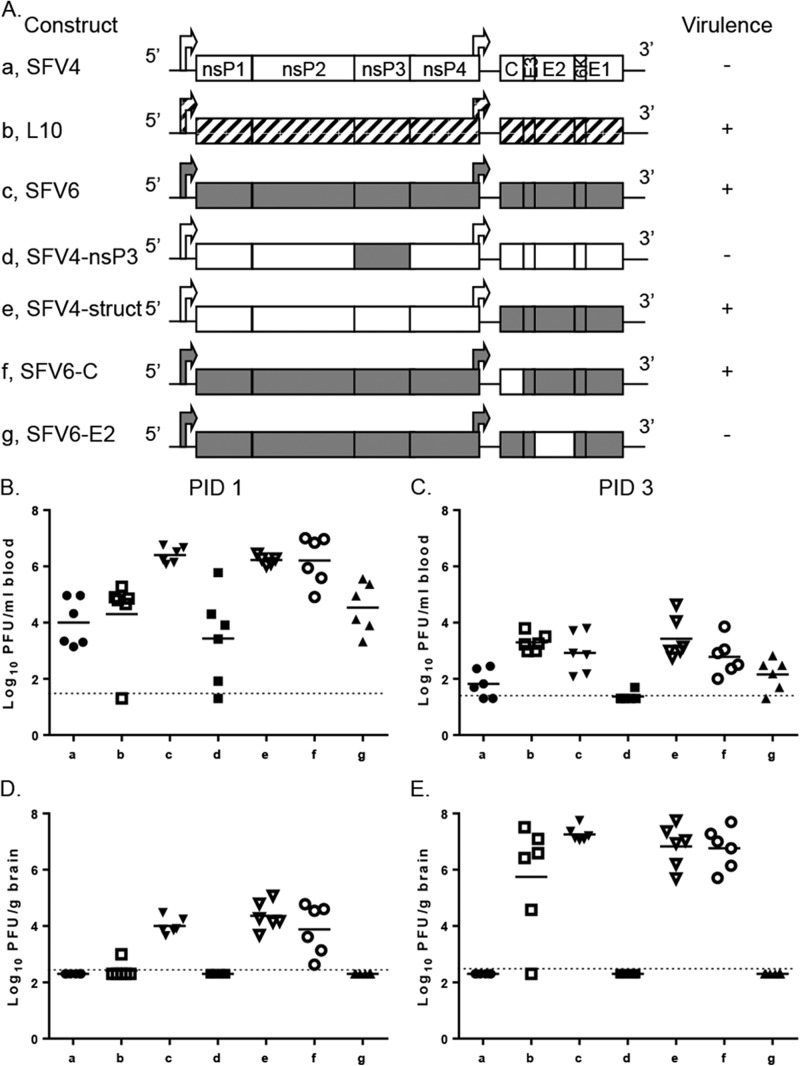
SFV variants vary in virulence, viremia, and brain titer following i.p. inoculation of adult mice. (A) Schematic representation of the genomes of SFV L10 and used SFV constructs. These viruses (5,000 PFU) were i.p. inoculated into 4- to 5-week-old BALB/c mice. Groups (*n* = 8) were monitored for survival for up to 2 weeks. Virulence (indicated by + or −) was defined as described in Materials and Methods. Virus titers in groups (*n* = 6) in the blood (B and C) and brain (D and E) were determined by plaque assay at PID 1 and 3. Each symbol represents an individual mouse. The line depicts the mean titer for each group. The dotted line depicts the limit of detection. Blood and brain virus titers for viruses b to g were compared by Mann-Whitney test to those for SFV4 (a) and SFV6 (c). In panel B, results of viruses c, e, and f were statistically significantly different from those for SFV4. Results for SFV6 also were significantly different from those for viruses b, d, and g. In panel C, titers of viruses b, c, e, and f were statistically significantly different from those for SFV4. SFV6 also was significantly different from d and g. In panel D, results for viruses c, e, and f were statistically significantly different from those for SFV4. SFV6 also was significantly different from b, d and g. In panel E, results for viruses b, c, e, and f were statistically significantly different from those for SFV4. SFV6 also was significantly different from d and g.

**FIG 2:**
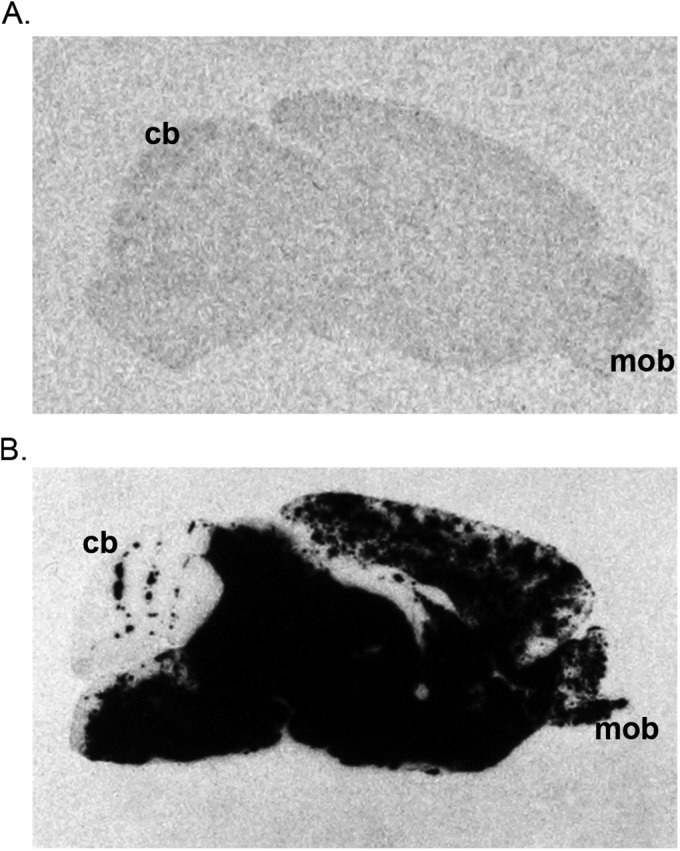
L10 disseminates more efficiently in the mouse brain following i.p. inoculation than SFV4. Representative autoradiographic images illustrating the distribution of virus RNA-positive cells (black) in parasagittal sections of the brains of 4- to 5-week-old BALB/c mice 72 h after i.p. inoculation with SFV4 (A) or L10 (B). *In situ* hybridization using 35S-labeled riboprobe complementary to the viral genomic RNA. For each of six mice sampled at each time point, three sections from each of three areas of the brain were studied; the images shown are representative of each group of mice. All brains are in the same orientation; the main olfactory bulb (mob) and the cerebellum (cb) demonstrate the rostro-caudal orientation. Following i.p. inoculation, no virus-positive cells were observed in mice inoculated with SFV4. In contrast, in mice inoculated with L10, virus-positive cells were identified throughout the brain.

To confirm that SFV4 and L10 differ in their levels of virulence, viremia, and neuroinvasive ability, groups of BALB/c mice were inoculated i.p. with 5,000 PFU virus and observed for up to 2 weeks or sampled at PID 1 and 3 to determine levels of infectious virus in the blood and the brain (Fig. 1). In this and in some subsequent experiments, levels of infectious virus were determined by both plaque assay and by quantitative real-time PCR for both inoculated stock viruses and tissues sampled. The two assays showed good correspondence for all viruses; therefore, only infectivity titers are shown in this and in subsequent figures. Consistent with previous studies, mice infected with L10 reached clinically defined endpoints by PID 5 while SFV4 was avirulent. Levels of viremia for the two viruses were equivalent at PID 1 but significantly higher for L10 than SFV4 at PID 3. Virus was detectable in the brain at PID 1 in only 1/6 L10-infected mice and none of the SFV4-infected mice. At PID 3, virus was detectable in the brains of 5/6 L10-infected mice but none of the SFV4-infected mice. To further investigate this, sagittal brain sections from all mice sacrificed at PID 3 were screened for virus RNA by *in situ* hybridization; 9 sections were screened for each mouse brain. No virus was detected in the brains of mice infected with SFV4. In contrast, many L10-positive cells were detected distributed throughout the brains (Fig. 2). These results confirm that following i.p. inoculation of 5,000 PFU L10, but not SFV4, is neuroinvasive.

### There are 6 residue differences between L10 and SFV4 encoded proteins.

To determine the genetic differences that dictate the neuroinvasive ability of these two SFV strains, the biological stock of L10 was sequenced using high-throughput sequencing and the consensus sequence was aligned to that of AY112987, an L10 sequence from a stock of virus from Trinity College Dublin. There were 18 residue differences, predominantly in nsP2 (data not shown). Our L10 consensus sequence then was compared to the published sequence of SFV4 (KP699763). Twelve nucleotide differences were identified (Table 1). These were located in the 5′ and 3′ untranslated regions (UTRs) and sequences encoding nsP1, nsP2, nsP3, capsid protein, E2, and E1. Six of these changes in coding regions led to a change in amino acid sequences of SFV-encoded proteins. Whereas phenotypic differences could be conferred by nucleotide changes that do not affect amino acid sequence, it is more likely that one or more of the six amino acid residue changes are responsible for the phenotypic difference in neuroinvasion. These six amino acid residue changes were investigated further.

**TABLE 1:** Genetic differences identified between the sequences of L10 and SFV4^*a*^

Location in genome	Position in SFV4		Amino acid residue(s) in SFV4-L10
	Nucleotide	Amino acid	
5′UTR	59		
nsP1	1627	514	V
nsP2	2485	800	L
nsP3	4236	1384	E-A
Capsid	7488	22	P
	7609	63	R-G
	7677	85	N-K
E2	8530	370	V-I
	8732	437	K-T
	8905	495^*b*^	K-E
E1	10923	1167	V
3′UTR	11333		

a Nucleotide and amino acid residue numbering is according to the published sequence of SFV4 (GenBank accession number KP699763). Amino acid residue numbering is from the start of the replicase and structural ORFs for the nsPs and the structural proteins. The 5′ untranslated region and replicase numbering also correspond to the published L10 reference sequence (AY112987). However, in contrast to the L10 reference sequence, the structural ORF and 3[prime] untranslated sequences shown are shifted by three nucleotides or one amino acid residue.

b Amino acid residue corresponds to residue number 162 in the E2 glycoprotein.

### Incorporation of the six nonsynonymous changes into SFV4 produces a fully virulent virus, SFV6.

To investigate whether the six nonsynonymous nucleotide changes identified determined neuroinvasion, all six were incorporated into the SFV4 molecular clone. This new molecular clone and the virus derived from it were designated SFV6 (GenBank accession number KT009012; Fig. 1). The amino acid sequence of proteins encoded by SFV6 is identical to the consensus amino acid sequence of proteins encoded by L10. It also was observed that relative to SFV4, SFV6 had a large plaque size on BHK-21 cells. Nevertheless, in BHK-21 cells SFV6 and SFV4 (as well as other recombinant viruses described below) grew to similar titers, and no significant differences in growth curves were observed.

As with L10, i.p. inoculation of 5,000 PFU of SFV6 into BALB/c mice resulted in all mice reaching clinically defined terminal endpoints by PID 5 (Fig. 1A). Subsequently, groups of BALB/c mice were i.p. inoculated (5,000 PFU) with SFV6, and virus titers in blood and brain were determined on PID 1 and 3 and compared to those obtained previously for L10 and SFV4 (Fig. 1). Compared to SFV4, SFV6 reached higher titers in the blood on both days and, unlike SFV4, was detected in the brain. SFV6 also produced a higher viremia on PID 1 than L10. Furthermore, unlike L10, SFV6 was detected in the brains of all infected mice at PID 1. These mice were not perfused; therefore, the virus detected in the brain probably reflects differences in blood content. By PID 3 there was no significant difference in blood or brain titers of L10 and SFV6. In conclusion, one or more of the six amino acid residue differences between SFV4- and L10/SFV6-encoded proteins determines viremia, neuroinvasion, and virulence.

### Phenotypic differences between SFV4 and SFV6 map to E2 region.

To further investigate the effect of these six nonsynonymous differences on phenotype, the corresponding substitutions were introduced, individually or in combination, into the SFV4 or SFV6 molecular clones (Fig. 1). Virus titers in the blood and brain were determined on PID 1 and 3 following i.p. inoculation (5,000 PFU) (Fig. 1B to E). The viremias of SFV4 and SFV4-nsP3, in which the nsP3 region of SFV4 was replaced with that of SFV6, were not significantly different, and neither of these viruses was detected in the brain. Consistent with this, SFV4-nsP3 also remained avirulent. This indicated that E1384A substitution in nsP3 (Table 1) did not determine neuroinvasion. In contrast, relative to SFV4, significantly higher titers of SFV4-struct, in which the structural region of SFV4 was replaced with that of SFV6, were detected in the blood, and this virus was detected in the brain (6/6 mice). In addition, SFV4-struct was virulent. This comparison indicated that one or more of the amino acid residue differences in the structural polyprotein determine the level of viremia and neuroinvasion.

Replacing the capsid protein of SFV6 with that of SFV4 (SFV6-C) did not have any significant effect on the neuroinvasive phenotype of the virus (as determined by brain virus titers), nor did it make the virus avirulent. In contrast, when the E2 envelope glycoprotein of SFV6 was replaced with that of SFV4 (SFV6-E2), the phenotype observed was similar to that of SFV4, indicating that the phenotypic differences in viremia, neuroinvasion, and virulence were determined by E2. These E2 differences also were responsible for the difference in BHK-21 plaque phenotype (data not shown).

### The biological stock of L10 is heterogeneous.

**FIG 3:**
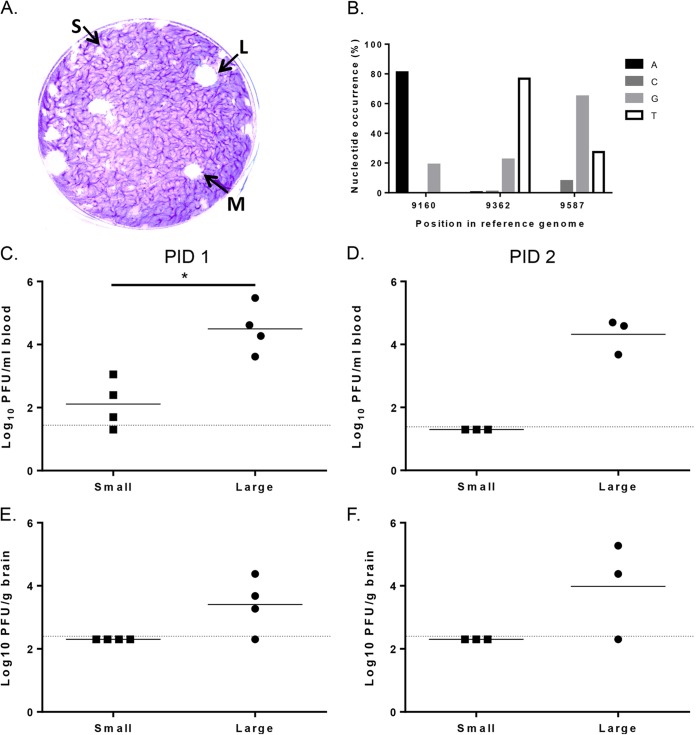
L10 is a heterogeneous stock containing viruses with three different plaque phenotypes. (A) Plaque assay of L10 on BHK-21 cells using an agar overlay. Small (S), medium (M), and large (L) plaques are labeled. (B) Three SNPs were identified (Piledriver) in E2 of the L10 high-throughput sequencing data. The nucleotide position in the reference genome and the occurrence of each of the four bases as a percentage are shown. Coverage was >10,000. The plaque-purified small and large plaque viruses from the biological stock of L10 (5,000 PFU) were i.p. inoculated into groups (*n* = 3 to 4) of 4- to 5-week-old mice, and at 1 and 2 days postinfection blood (C and D) and brain (E and F) virus titers were determined by plaque assay. PBSA was included as a control, and no virus was detected. Each symbol represents an individual mouse. The line depicts the mean titers for each group. The dotted line depicts the limit of detection of the assay. Significant difference is shown by an asterisk (*P* < 0.05, Student's *t* test). Groups on PID 2 were too small for statistical analysis.

Although the amino acid sequence of SFV6-encoded proteins matched the consensus amino acid sequence of L10 proteins, inoculation of these two viruses into mice resulted in an early difference in phenotype with a higher viremia for SFV6 than for L10 at PID 1. The stock of L10 used in these studies was isolated from a pool of mosquitoes in Uganda (10) and underwent various recorded passages *in vitro* and *in vivo* before being stored in the freezer for over 30 years. This stock then was used to generate new virus stocks by passage on BHK-21 cells. These stocks subsequently were used to study the acute encephalitis generated by L10 virus (1). As far as we can ascertain, this L10 stock was never plaque purified. Indeed, on BHK-21 cells the stock of L10 produced a mixed plaque phenotype of small, medium, and large plaques (Fig. 3A). The small-plaque virus had a plaque diameter of <1 mm, medium plaques were 2 to 3 mm, and large plaques were 3 to 4.5 mm. This indicates that the L10 stock contains at least three different genotypes. To determine the genetic basis for these different phenotypes, viruses from six plaques of each size (small, medium, and large) were purified and their E2 genes sequenced. Compared to SFV4, all plaque-purified viruses had nonsynonymous differences in E2 at nucleotide positions 8530, 8732, and 8905 (all present in SFV6) (Table 1). Both the medium- and the large-plaque virus differed from the consensus sequence of L10 at nucleotide position 9587, which is part of the codon for amino acid residue 389 of E2 (alanine in isolated viruses, valine in consensus sequence). The medium-sized plaque-purified virus had additional synonymous nucleotide changes at positions 8751 and 8959. In the small-plaque viruses, only one nucleotide difference at position 9160 led to a change of glutamic acid (L10 consensus sequence) to lysine at position 247 of E2 protein (E247K).

To determine whether the E2 nucleotide changes identified in the plaque-purified viruses were present in the high-throughput sequencing data, L10 E2 reads were mapped to the L10 consensus genome using Bowtie2-2.10 and SAMtools and fed into Piledriver (17, 18). E2 SNPs were identified at nucleotide positions 9160, 9362, and 9587 (Fig. 3B). Nucleotides at positions 9160 and 9587 were those that resulted in nonsynonymous differences between small- and large-plaque viruses. The SNP at position 9362 was not present in the sequences data of the plaque-purified viruses, and synonymous differences in positions 8751 and 8959 found in the case of viruses with medium plaque size did not qualify as SNPs. In summary, these data demonstrated that L10 is a heterogeneous stock containing at least three major variants of E2 coding sequences. Of the identified changes, the nucleotide at codon 247 of E2 changed the charge of the encoded amino acid residue from negative to positive.

To investigate phenotypic differences between the large- and small-plaque-purified L10 viruses, groups of BALB/c mice were inoculated i.p. with the plaque-purified viruses and titers in the blood and the brain determined on PID 1 or 2 (Fig. 3C to F). Titers of the large-plaque virus in the blood at PID 1 were significantly higher than those of the small-plaque virus. On PID 2, the large-plaque virus still was present in the blood, while the small-plaque virus was undetectable. It was not possible to detect the small-plaque virus in the brain on either PID 1 or 2. In contrast, large-plaque virus was detected in the brain on PID 1 and the titer had increased by PID 2, at which point it was in the same order of magnitude as that in the blood, indicating replication in the brain. In conclusion, E2 247 may be a determinant of viremia and neuroinvasion.

### Residues 162 and 247 of E2 are determinants of viremia, neuroinvasion, and virulence.

**FIG 4:**
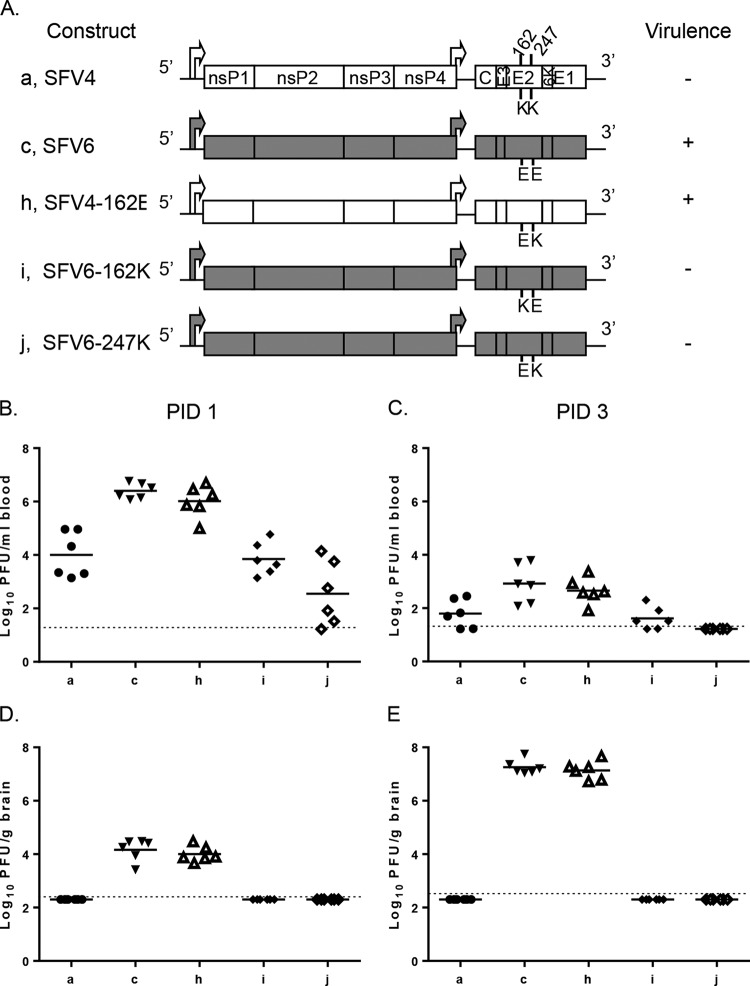
SFV variants with amino acid residue differences in E2 vary in virulence, viremia, and brain titer following i.p. inoculation of adult mice. (A) Schematic representation of SFV constructs. These viruses (5,000 PFU) were i.p. inoculated into 4- to 5-week-old mice. Groups (*n* = 8) were monitored for survival for up to 2 weeks. Virulence (indicated by + or −) was defined as described in Materials and Methods. Virus titers in groups (*n* = 6) in the blood (B and C) and brain (D and E) were determined by plaque assay at PID 1 and 3. Each symbol represents an individual mouse. The line depicts the mean titers for each group. The dotted line depicts the limit of detection. In panels B to E, blood and brain virus titers for viruses c, h, i, and j were compared to those for SFV4 (a); results for c and h were statistically significantly different (*P* ≤ 0.05 by Mann-Whitney test). In addition, blood and brain virus titers for viruses h, i, and j were compared to those for SFV6 (b); results for i and j were statistically significantly different (*P* ≤ 0.05 by Mann-Whitney test).

The ability of SFV4 and SFV6 to replicate in the periphery and enter the brain was determined by one or more of the three amino acid residue differences in E2. Of these, position 162 has been described previously as a molecular determinant of SFV virulence (22), and position 247 affects L10 plaque phenotype, viremia, and neuroinvasion (Fig. 3). To directly test the importance of these two amino acid residues, three new viruses were engineered: SFV4-162E, where the lysine (K) at E2 position 162 was changed to glutamic acid (E) (large plaque phenotype on BHK-21 cells), SFV6-162K, containing the reciprocal change, and SFV6-247K, where glutamic acid 247 in E2 of SFV6 was changed to a lysine (both small-plaque phenotypes on BHK-21 cells) (Fig. 4A).

Groups of mice were inoculated i.p. with 5,000 PFU of these viruses (and the parental viruses as controls) and monitored for survival. Alternatively, virus titers in blood and brain were determined on PID 1 and 3 (Fig. 4B to E). As in the previous experiment, SFV4 was detected in the blood but not in the brain and was avirulent, while SFV6 was at higher titers in the blood, was detectable in the brain, and was virulent. SFV4-162E was virulent, produced a significantly higher viremia than SFV4 on both PID 1 and 3, and was detected in the brain. There were no significant differences in the titers between SFV4-162E and SFV6 in the blood or brain, indicating that the glutamic acid residue at E2 162 increases viremia and is a determinant of neuroinvasion. Both SFV6-162K and SFV6-247K were avirulent, like SFV4. Levels of these viruses in the blood were lower than that for SFV6 on both PID 1 and 3, and these viruses were not neuroinvasive. This indicated that a lysine residue in position 162 or 247 of E2 reduces blood virus titer, neuroinvasion, and virulence.

### Amino acid residues in positions 162 and 247 in E2 affect SFV replication in the mouse brain following i.c. inoculation.

**FIG 5:**
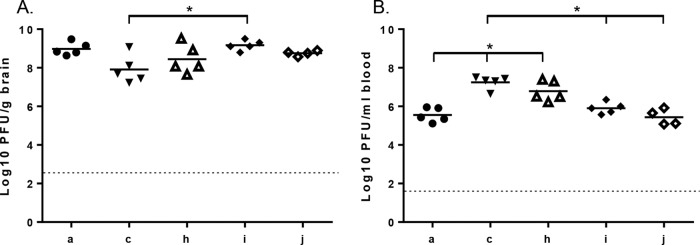
SFV replication in the brain and the periphery following i.c. inoculation. SFV4 (a), SFV6 (c), SFV4-162E (h), SFV6-162K (i), and SFV6-247K (j) (1,000 PFU) were i.c. inoculated into groups (*n* = 5) of 4- to 5-week-old mice. At 30 hpi, mice were euthanized and virus titers in the brain (A) and the blood (B) were determined by plaque assay. No virus was detected in either the blood or the brain of one mouse in the SFV6-247K group; therefore, this individual was removed from the analysis. Each symbol represents an individual mouse. The line depicts the mean titer of each group. The dotted line depicts the limit of detection. In panel A, brain titers of SFV4 (a) and SFV6 (c) were compared to each other and to those of viruses c, h, i, and j; results for SFV6 were significantly different from those for virus i (*, *P* ≤ 0.05, Mann-Whitney test). The other titers were not statistically different from each other. In panel B, blood titers of SFV4 (a) were compared to those of viruses c, h, i, and j; those of c and h were significantly different (*, *P* ≤ 0.05, Mann-Whitney test). In addition, SFV6 (c) titers were compared to those of h, i, and j; those of i and j were significantly different (*, *P* ≤ 0.05, Mann-Whitney test).

To determine whether the amino acid residues 162 and 247 of E2 affect entry into and/or replication within the brain, SFV4, SFV6, SFV4-162E, SFV6-162K, and SFV6-247K were inoculated i.c. into groups of BALB/c mice, and titers of infectious virus in brain were determined at 30 hpi (Fig. 5A). Both SFV4 and SFV6 replicated in the brain; indeed, SFV4 replicated to higher titers than SFV6. This effect was reduced by replacing lysine 162 in E2 with glutamic acid. Conversely, the efficiency of SFV6 replication in the brain was increased by replacing glutamic acid residue 162 or 247 of E2 with a lysine residue.

Titers of infectious virus content were determined for blood samples from the mice inoculated i.c. (Fig. 5B). Similar to the i.p. infection (Fig. 1 and 4), it was found that SFV4, SFV6-162K, and SFV6-247K replicated to significantly lower titers than SFV6 and SFV4-162E. In conclusion, the level of viremia for SFV4 was lower than that for SFV6, but both viruses had the ability to replicate in the brain, with SFV4 (and other avirulent mutants) replicating even slightly better than SFV6 (and virulent mutants). These phenotypes correlated with the amino acid residues at E2 positions 162 and 247, indicating that charge (positive or negative) at this position has a crucial impact on the *in vivo* properties of virus.

### E2 162 affects the integrity of a model BBB.

**FIG 6:**
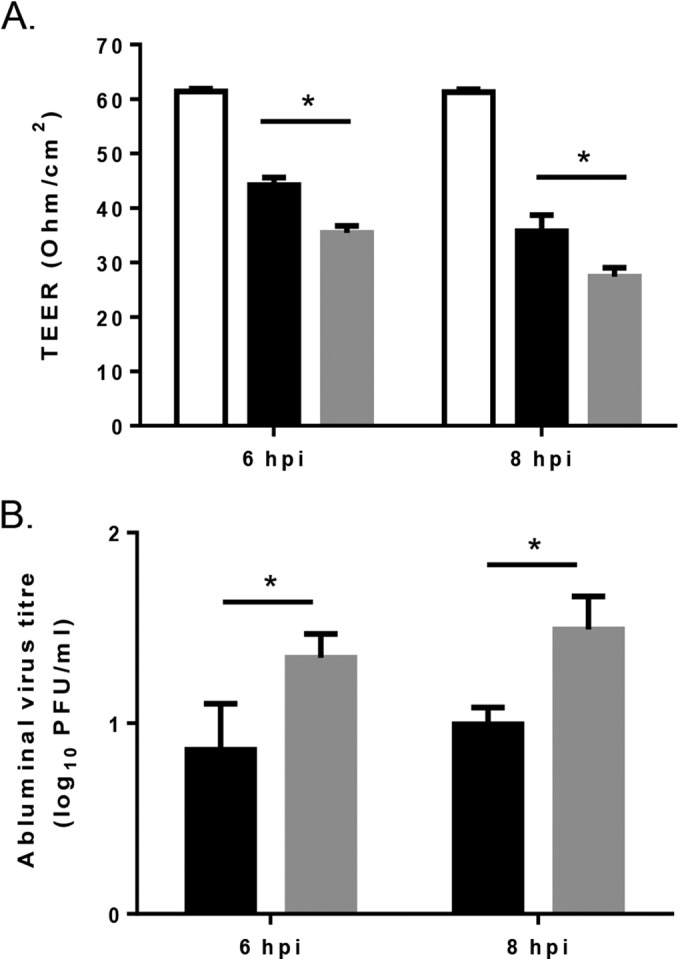
Effect of amino acid residue position 162 of E2 on infection of the human *in vitro* BBB model. (A) SFV6 (black bars) and SFV6-162K (gray bars) were placed on the luminal side of the BBB model (MOI of 0.1), and BBB permeability was assessed by measuring the transendothelial electrical resistance (TEER) at 6 and 8 hpi. The permeability of uninfected control cells was included for comparison (white bars). (B) Virus titers on the abluminal side measured by plaque assay. Results are mean values ± standard deviations (*n* = 3). Significant difference is shown by an asterisk (*P* < 0.05, Student's *t* test).

In the preceding studies, neuroinvasion correlated with viremia, with SFV4, SFV6-162K, and SFV6-247K having a low-titer viremia and no virus in the brain and SFV6 and SFV4-162E having a high-titer viremia and virus in the brain. To investigate whether neuroinvasion was related solely to the level of viremia or also to the ability of virus to cross the BBB, the ability of different viruses to cross a model *in vitro* BBB was determined. The BBB was composed of primary astrocytes and HBEC-5i cells. SFV6 or SFV6-162K was added to the luminal side of the BBB, and the integrity of the BBB TEER was measured at 6 and 8 hpi. Infection with both strains led to a significant drop in TEER, indicative of increased permeability. However, the SFV6-162K infection led to a significantly greater reduction in TEER compared to that with SFV6 infection at both 6 and 8 hpi (Fig. 6A). Virus titers on the abluminal side were determined at 6 and 8 hpi by plaque assay. Significantly more virus was detected on the abluminal side following infection with SFV6-162K than after infection with SFV6 at both 6 and 8 hpi (Fig. 6B). In conclusion, although SFV6-162K generated a lower viremia than SFV6, it had a larger effect on the integrity of the BBB.

### Serial passage of SFV6 in tissue culture selects for positively charged amino acid residues in E2.

Selection for positively charged amino acid residues in virus envelope glycoproteins by passage in cell culture has been reported for Sindbis virus (SINV) (23). To determine whether the lysine at E2 162 in SFV4 and the lysine at 247 in the small-plaque variant of L10 is a tissue culture adaptation, four biological replicates of SFV6 (glutamic acid at E2 162 and 247) were serially passaged on BHK-21 cells at a low MOI (0.01 and 0.001). Subsequently, the region of genome encoding E2 from each replicate was reverse transcribed and PCR amplified at passage 5 and passage 10 (Table 2). By passage 5, a positively charged amino acid residue in E2 had been selected in 3/8 passaged viruses. In two of these samples the amino acid residue change was E247K. By passage 10, 6/8 of the passaged viruses had acquired positively charged lysine residues at either position 212 or 247 of E2.

**TABLE 2:** Serial passaging of SFV6 in BHK-21 cells selects for positive amino acid residues in E2

MOI and biological repeat	Residue at passage no.^*a*^:	
	5	10
0.01		
1		E247K
2		
3	E247K	E247K
4		E247K
0.001		
1		
2	N212K	N212K
3		N212K
4	E247K	E247K

a Amino acid residue in SFV6 at passage 0 followed by the amino acid residue position in E2 and then the amino acid residue in SFV6 at the corresponding passage.

### Amino acid residues 162 and 247 are predicted to be on the surface of the alphavirus spike.

**FIG 7:**
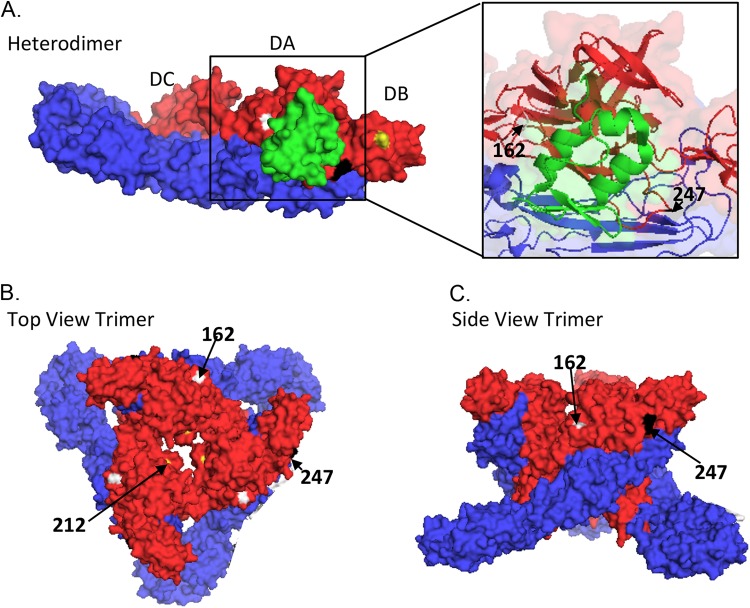
Location of amino acid residues 162, 212, and 247 in the alphavirus E1/E2 heterodimer and trimer. (A) Surface view of the alphavirus E1 (blue) and E2 (red) heterodimer. E3 is shown in green. E2 domain A (DA), E2 domain B (DB), and E2 domain C (DC) are labeled. The E1/E2 heterodimer was modeled based on Protein Data Base (PDB) code 3N42 and edited in PyMol (http://www.pymol.org). An enlargement of the boxed section in panel A showing the secondary structure is on the right. amino acid residues 162 (white), 212 (yellow), and 247 (black) are labeled (arrows). The top view (B) and side view (C) as shown of the alphavirus trimer based on the cryo-EM of PDB entry 2XFC.

Amino acid residues E2 162, 212, and 247 were located on the three-dimensional (3D) structure of alphavirus E1/E2 heterodimer and trimer using the software PyMol (http://www.pymol.org) (Fig. 7). Alphavirus virions are composed of a genomic RNA encapsulated by the nucleocapsid, which is surrounded by an envelope containing 240 E1/E2 heterodimers in 80 trimeric E1/E2 spikes (24). The structure of E1/E2 heterodimer of chikungunya virus (CHIKV) was determined previously by X-ray crystallography (PDB code 3N42), and the structure of the CHIKV trimer was predicted based on cryoelectron microscopy of SFV virions (PDB code 2XFC [24]). Based on the structure of E1/E2 heterodimer, amino acid residues 162 and 247 were predicted to lie within the acid-sensitive region (ASR) in domain A of E2. Positions 162, 212, and 247 appeared to be on the surface of the E1/E2 heterodimer. When all positions were mapped onto the trimer, they clearly lay on the surface of the spike. In alphaviruses, E2 mediates receptor binding and receptor-mediated endocytosis. Therefore, positions 162, 212, and 247 of E2 are probably available to bind proteins and other molecules, such as cell surface receptors.

### Amino acid residues at positions 162 and 247 of E2 modulate virus binding to heparan sulfate.

**FIG 8:**
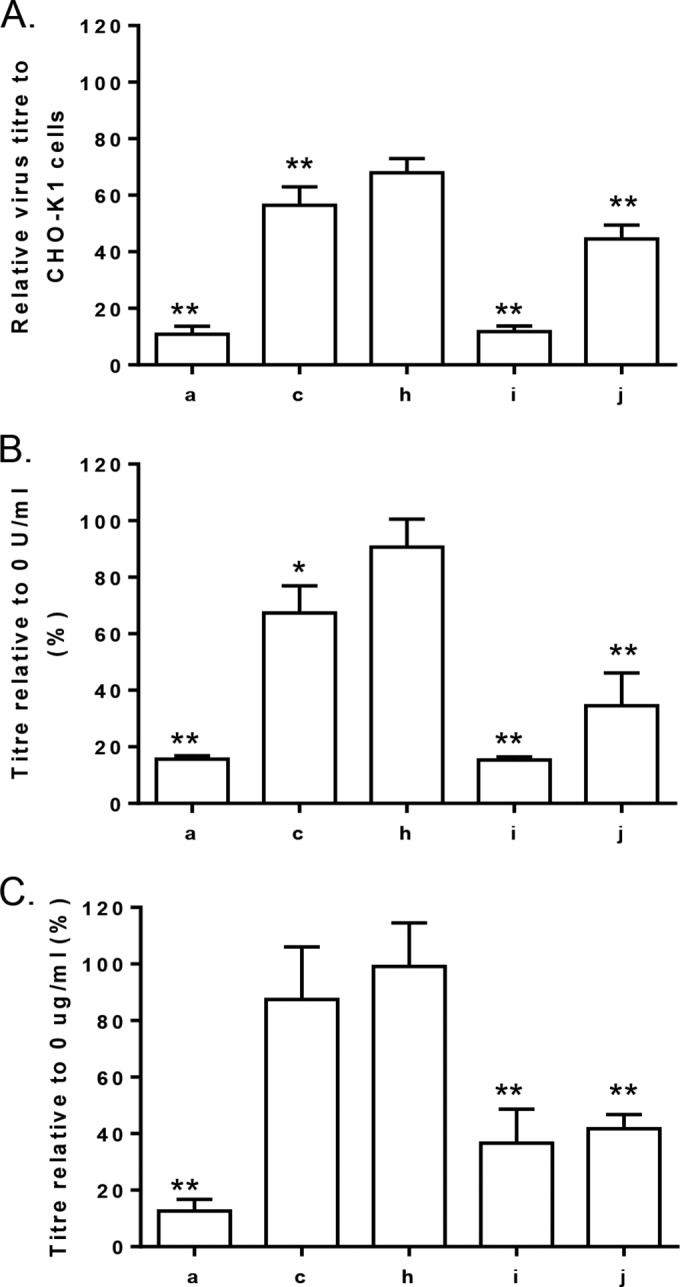
Amino acid residues 162 and 247 of E2 affect binding to heparan sulfate. (A) Titers of SFV4 (a), SFV6 (c), SFV4-162E (h), SFV6-162K (i), and SFV6-247K (j) on CHO-K1 cells and the derivative cell line pgsA-745, which lack GAGs on their surfaces, were determined by plaque assay. Virus titers on CHO-K1 cells were set to 100%, and virus titers on pgsA-745 cells are displayed relative to this. Significant difference from the CHO-K1 cells is shown by two asterisks (*P* < 0.01, Student's *t* test). Each bar is the mean of replica samples from three experiments. (B) Virus titers were determined on BHK-21 cells that were pretreated with 0 or 0.5 U/ml of heparinase I. Virus titers on BHK-21 cells pretreated with 0 U/ml of heparinase I were set to 100%, and virus titers on BHK-21 cells pretreated with 0.5 U/ml of heparinase I are displayed relative to this. Significant difference from the control cells is shown by one or two asterisks (*P* < 0.05 or *P* < 0.01, respectively; Student's *t* test). Each bar is the mean of replica samples from three experiments. (C) Viruses were incubated with 0 or 10 μg/ml of heparin for 30 min, and then titers of virus were determined on BHK-21 cells. Virus titers after incubation with 0 μg/ml of heparin were set to 100%, and virus titers were compared to those of SFV4 or SFV6 infections. Significant difference from the control cells is shown by two asterisks (*P* < 0.01, Student's *t* test). Each bar is the mean of replica samples from three experiments.

Selection for positively charged amino acid residues in SINV E2 promotes binding to the ubiquitously expressed negatively charged glycosaminoglycan (GAG) heparan sulfate (HS) (23). In other alphaviruses, including Ross River virus (RRV), CHIKV, eastern equine encephalitis virus (EEEV), and VEEV, changes in E2 amino acid in similar locations on the glycoprotein trimer to those in SFV (Fig. 7) also affect virus binding to HS (25–28). SFV4 has been demonstrated previously to bind to heparin (29). Therefore, we hypothesize that SFV4, SFV6, SFV4-162E, SFV6-162K, and SFV6-247K differ in their interaction with HS. To investigate this, three experiments were carried out (Fig. 8).

First, to determine if the presence of GAGs on the surface of cells affects virus entry into cells, stocks of these viruses were plaque titrated on CHO-K1 and their derivative pgsA-745 cells, which are deficient in GAGs. Formed plaques were counted at 72 hpi. All five viruses formed fewer plaques on pgsA-745 cells than CHO-K1 cells. However, the efficiency of plaque formation of SFV4 was reduced by approximately 90%, while that of SFV6 was reduced by only 40% (Fig. 8A). Swapping the amino acid residue 162 in E2 reversed the phenotype: the number of plaques made by SFV4-162E on pgsA-745 cells was 40% of that on CHO-K1 cells, whereas SFV6-162K plaques were reduced by 90%. In this experiment, SFV6-247K displayed an intermediate phenotype and made approximately 55% fewer plaques on pgsA-745 cells (Fig. 8A).

Second, to investigate whether the lack of HS on the surface of cells affects virus entry on other cell types, BHK-21 cells were pretreated with heparinase I, which cleaves the linkages between hexosamines and O-sulfated iduronic acids found in HS and heparin. The viruses then were added to cells for 30 min, followed by an agarose overlay, and formed plaques were counted at 72 hpi. When the number of plaques made on treated and nontreated cells were compared, it was found that heparinase I treatment reduced plaque formation by both SFV6 and SFV4 (Fig. 8B). However, consistent with results of previous experiments, plaque formation by SFV4 was affected much more prominently than that of SFV6; data obtained for SFV4-162E, SFV6-162K, and SFV6-247K confirmed that this phenotype again segregated according to the amino acid residues at positions 162 and 247 of E2.

Finally, a competition assay was carried out to investigate whether SFV4, SFV6, SFV4-162E, SFV6-162K, or SFV6-247K differentially binds to heparin. These viruses were preincubated with heparin or BSA as a control for 30 min on ice and then added to BHK-21 cells for 30 min on ice and covered with an agarose overlay. The number of formed plaques was determined at 72 hpi. Again, it was found that compared to BSA treatment, the preincubation with heparin reduced plaque formation efficiencies in a manner very similar to that observed in previous experiments. Thus, exposure to heparin reduced the number of plaques made by SFV4 more than that in the case of SFV6 (Fig. 8C). Again, analysis of SFV4-162E, SFV6-162K, and SFV6-247K confirmed that the phenotype segregated according to amino acid residues 162 and 247 of E2.

Taken together, the highly consistent data from these three experiments clearly demonstrated that SFV4 and SFV6 had differential binding to HS and heparin and that this was determined by the charge on the amino acid residues at positions 162 and 247 of E2. A positively charged lysine residue at position 162 or 247 in E2 of SFV augmented virus entry into cells through greater binding to GAGs such as HS; in the absence of such a molecule, the entry (most likely because of reduced efficiency of binding to cells using alternative molecules) of SFV4, SFV6-162K, and SFV6-247K was more seriously affected than entry of SFV6 or SFV4-162E.

## DISCUSSION

Following low-dose i.p. inoculation into adult mice, strains of SFV differ in their ability to induce encephalitis. The L10 strain is rapidly and efficiently neuroinvasive, generating a panencephalitis and killing mice within 6 days. In contrast, the closely related SFV4 is rarely neuroinvasive and has low virulence. In this study, we demonstrate that lysine or glutamic acid residues at position 162 or 247 in the SFV E2 glycoprotein affects the level of viremia, the efficiency of neuroinvasion, and neurovirulence in mice, and that this correlates with binding to HS.

The consensus sequence of an archived stock of L10 of known provenance was determined by high-throughput sequencing. Differences resulting in amino acid residue changes relative to those of SFV4 were engineered into the SFV4 icDNA to generate a new icDNA, designated SFV6. Virus produced from the SFV6 clone inoculated into mice generated a high-titer viremia and was efficiently neuroinvasive. For the first time, this provides a molecular clone (icDNA) which generates SFV with the same phenotype as the L10 and prototype biological strains.

There were several single-amino-acid-residue changes between the proteins encoded by SFV4 and SFV6. Generation of recombinant viruses showed that the genetic locus determining neuroinvasion mapped to E2. That a single-amino-acid change in E2 can affect neurovirulence in mice has been observed previously for SINV, VEEV, and EEEV (26, 30–32). In the case of SFV, reciprocal changes of lysine and glutamic acid at E2 162 demonstrated that this difference alone was a strong determinant of the level of viremia, neuroinvasion, and virulence. SFV4 and SFV6-162K had a small-plaque phenotype, generated low-titer viremia, and were not detected in the brain. SFV6 and SFV4-162E had a large-plaque phenotype, generated high-titer viremia, and were detected in the brain. Interestingly, in an earlier study, infection of pregnant BALB/c mice with high-dose SFV4 was attenuated if E2 162 was changed from lysine to glutamic acid (9, 22). Brain virus titers were not reported in this study; charge at E2 162 may affect the ability of virus to cross the placenta.

The archived L10 stock used in this study had been passaged in chicken embryo fibroblasts and then stored frozen for 32 years. It had not been plaque purified and contained three plaque variants and at least three different E2 genotypes with either a negatively charged glutamic acid or a positively charged lysine at amino acid 247. These gave rise to large (glutamic acid) or small plaques (lysine) and were virulent or avirulent, respectively. The presence of quasispecies within the L10 stock of various virulence highlights the importance of molecular clones in research and, therefore, the usefulness of SFV6. Furthermore, it demonstrates the importance of minimal *in vitro* passaging of WT viruses before engineering a molecular clone.

Large-plaque, high-titer viremia, virulent SFV6, and the large-plaque variant of L10 have negatively charged glutamic acid at E2 162 and E2 247. In SFV4, E2 162 is a positively charged lysine. In the small-plaque variant in the historic L10 stock, E2 247 is also a positively charged lysine. Both SFV4 and the small-plaque variant were low-viremia, low-virulence viruses. SFV6 viruses in which glutamic acid at E2 162 or 247 was changed to lysine also were converted into small-plaque, low-level-viremia, nonneurovirulent viruses, confirming that these E2 charge changes each were sufficient to cause this major change in phenotype. Changes to positively charged amino acid residues in the E2 proteins of other alphaviruses, selected by passaging or engineered by site-directed mutagenesis, also are associated with reduction in peripheral virus titers, rapid clearance from the blood, and low virulence (23, 26, 33). This correlates with the ability of these viruses to interact with GAGs, more specifically with HS. In addition to alphaviruses, several other viruses have been demonstrated to utilize HS binding to enter cells in culture, including flaviviruses, picornaviruses, arteriviruses, herpesviruses, and lentiviruses (34–40).

GAGs are ubiquitously expressed, negatively charged polysaccharides found on the surface or in the extracellular matrix of both vertebrate and invertebrate cells (41–43). In general, proteins interact with negatively charged HS via positively charged lysine and arginine residues. When proteins that bind to HS are injected intravenously, they are rapidly cleared from the circulation (44, 45). This clearance can be negatively affected by coinjecting with heparin or by injecting heparinase that digests HS on the tissue surface (44, 46). Alphaviruses that efficiently bind to HS most likely are cleared from the periphery by the same mechanism, resulting in a lower viremia. This may be due to HS-binding virus being absorbed in the liver, an organ rich in GAGs (26). In the present study, SFV containing lysine at either E2 162 or 247 was more reliant on HS for entry into CHO-K1 or BHK-21 cells than virus having glutamic acid at these positions. Furthermore, heparin competed more efficiently with HS for binding to SFV with a lysine residue at either E2 162 or 247. This is consistent with a recent finding that CHIKV with an E2 that interacts with GAGs does not disseminate efficiently to lymphoid tissues or efficiently stimulate inflammatory responses (47). Increased binding to HS is associated with decreased neurovirulence for VEEV and for the flavivirus Murray Valley encephalitis virus (26, 48).

When inoculated directly into the mouse brain, SFV with lysine at E2 162 or 247 replicated to a higher titer than SFV with glutamic acid at these positions. This is consistent with the previous finding that SFV4 with E2 162 lysine is more virulent than SFV4 with E2 162 glutamic acid when inoculated intranasally, a direct neural route to the CNS (9). Similarly, greater binding to HS is associated with increased neurovirulence in neonatal mice infected with SINV, in adult mice inoculated i.c. with SINV, and in adult mice inoculated i.c. with a natural North American strain of EEEV (31, 32, 49).

When the ability of SFV6 and SFV6-162K to pass across an *in vitro* model BBB was compared, SFV6-162K did so more efficiently than SFV6. Cells in culture have more GAGs on their surface, and the increased ability to cross the BBB by SFV6-162K may simply reflect this. It also could reflect a difference *in vivo*. For strains of HIV, the ability to cross an *in vitro* BBB correlates with efficiency of binding to HS (50, 51). For SFV, the ability to cross the *in vitro* BBB correlates with the ability to replicate in the brain, but not with the ability to generate a high-titer viremia. Selective pressures in the periphery and within the brain clearly are different and also may be different at the BBB. It is likely that for SFV, initiation of brain infection is determined both by plasma virus load and the efficiency with which virus traverses the BBB.

For other alphaviruses, including SINV, CHIKV, RRV, and EEEV, amino acids on E2 which affect binding to HS map to the apical surface of the CHIKV trimer (24, 27, 28). Similarly, the three SFV E2 residues (160, 212, and 247) identified in the current study mapped to the apical surface of the CHIKV trimer. In all cases these affected the electrostatic charge of the trimer and were associated with changes in virus virulence.

In this study, we engineered for the first time an SFV molecular clone, SFV6, that produces a virus with the same phenotype as that of SFV L10, providing a novel tool for future alphavirus research. Relative to virus derived from the existing SFV4 molecular clone, SFV6 had six amino acid differences. The differences in phenotype between these two viruses were determined by changes of charged amino acid residue, glutamic acid to lysine, on the surface of the E2 glycoprotein molecule. Another important determinant of phenotype was identified at E2 position 247; this also was either glutamic acid or lysine. Lysine facilitated the ability of virus to cross a model *in vitro* BBB and to replicate in the mouse brain. Glutamic acid increased the virus load in mouse plasma, neuroinvasion, and virulence. However, lysine in E2 promoted neurovirulence when SFV was inoculated i.c. These phenotypes correlated with the ability of a virus to bind GAGs.
